# Managing physician lipid management: a population wide, risk-based decision support approach

**DOI:** 10.1186/s13584-015-0032-9

**Published:** 2015-07-15

**Authors:** Lisa V. Rubenstein

**Affiliations:** VA HSR&D Center for the Study of Healthcare Innovation, Implementation and Policy, VA Greater Los Angeles Healthcare System, Sepulveda, CA USA; Department of Health Policy and Management, UCLA Fielding School of Public Health, Los Angeles, CA USA; VA QUERI Center for Implementation Practice and Research Support, VA Greater Los Angeles Healthcare System, Sepulveda, CA USA; Department of Medicine, VA Greater Los Angeles Healthcare System and UCLA Geffen School of Medicine, Los Angeles, CA USA; RAND Health, RAND Corporation, Santa Monica, CA USA

## Abstract

Successful implementation of clinical guidelines for preventing complications of dyslipidemias has been an ongoing challenge. The article by Vinker and colleagues in this journal investigates the results of implementing risk-based guidelines for LDL (Low Density Lipoprotein) management in comparison to the prior approach of using the same LDL cutoff for patients at all levels of risk. Results show LDL levels dropped across the primary care population using the new risk-based approach, suggesting that clinical decision aids that link to individual patient characteristics, rather than promoting a universal target for all, may provide a particularly strong stimulus for changing provider and patient behavior. Results also challenge healthcare organizations, providers and patients to learn more about the pathway from guidelines to clinical reminders and from reminders to lower LDL levels and better population health.

## Background

A recent study in this journal provides fascinating glimpses into the future of population-based guideline implementation, in addition to its implications for lipid management, for use of computer reminders, and for understanding healthcare provider behavior [1]. Undertaken by Vinker and colleagues, the study documents lipid level outcomes for the population of adults enrolled in a large managed care organization (Clalit Health Services, with a population of more than four million beneficiaries) after implementation of risk-based lipid management guidelines. Study data follows the health system’s transition from a prior one size fits all approach to the risk-based approach. Both the prior approach and the new approach were supported by computer-based clinical reminders.

## Risk-based guideline goals and implementation results

Unlike many studies of provider behavior, this study’s primary outcome is change in the target physiologic measure—the primary care population’s lipid levels (based on Low Density Lipoprotein or LDL)—rather than simply change in a process of care, such as the proportion of patients tested for LDL. Risk groups are identified based on electronically available clinical factors (e.g., heart disease, age, and smoking status). In the risk-stratified approach, reminders targeted higher risk patients for achieving more stringent goals. The new target for high risk patients is an LDL-C level <100, while for medium risk patients it is <130 and for low risk it is <160. These targets are based on guidelines from the National Cholesterol Education Program Adult Treatment panel III (ATP III) [2]. In the earlier approach, reminders targeted an LDL-C level of less than 100 across all risk groups. Study outcome results retrospectively applied the new guideline targets at both time periods, and showed significant improvement in ATP III LDL goal attainment in the later time period. Implementation of risk-stratified LDL management linked to improved outcomes for each risk group (high, medium and low). Results also showed increases in physician prescribing of high potency statins and a decrease in lower potency statins. As is often the case in studies of real-world implementation of scientific findings, however, the importance of study may rest as much with the questions it raises as with the answers it provides.

## Looking forward

One question implied by the study is what role the guidelines themselves had in shaping the study results. Given that the ATP III guidelines came out in 2002 and the risk-based computer reminder was implemented in Clalit Health Services (henceforth Clalit) only in 2011, study results are unlikely to be due to dissemination of the new guidelines. The structure of the ATP III guidelines, however, provided clear support for a risk-based approach, while the majority of existing clinical guidelines do not. While this study was not designed to test a specific causal pathway to the LDL outcomes, the study provides indirect evidence that risk-based guidelines may be more motivating to clinicians, perhaps because this type of guideline aligns better with clinical thinking and knowledge. This conclusion is supported by prior work using vignettes [3]. At the same time, advancing information technology has enabled the widespread implementation of nuanced, algorithm-based computer reminders that follow clinical guideline logic. Together, risk-based guidelines and the availability of computer decision support for implementing them can promote incorporation of information on differential risks and benefits [4] into routine clinical decision-making. Doing so may enable clinical reminders to achieve a closer match to clinical thinking as well as to the goals of personalized medicine.

Computer reminders have become a standard of practice for assisting providers in achieving guideline adherence. Prior to this study, an assumption might have been that physicians would respond best to the simplest reminder, such as the prior reminder targeting an LDL level of 100 for everyone. This study shows that a more complex reminder can be motivating to clinicians. Yet more complex reminders often require more computer “clicks”, and can add to the workload in primary care. A single extra click, for example, repeated over the number of patients, physicians, and reminders in primary care can add substantial cost. As reminders multiply, it will be critical to assess when complexity is helpful and when it is not, and to aim for the lowest workload approach that achieves clinical goals. This study did not report on human factors, such as time to complete, unclear instructions, or other usability characteristics. Is it time to require those implementing reminders to report on user acceptability and efficiency? My answer is yes; review of data on the experiences of physicians and teams as they use a new reminder should be an integral part of deciding whether and how to implement it.

A prior meta-analysis showed that healthcare provider-targeted interventions had a significant effect on patient medication adherence. Adding interventions targeted at improved integration of care or improved provider communication skills with patients had enhanced effects [5]. Clalit results on improved LDL levels, by definition, reflect physician and primary care team effects on patient adherence to either medications or lifestyle changes. They may also reflect independent changes in patient readiness to adopt LDL treatments, such as through greater public awareness. Future work could address additional questions about the paths from reminders to provider behavior change to patient behavior change. How did these providers transmit guideline recommendations to patients? What did providers tell high versus low risk patients with high LDLs? To what extent are physicians informing patients about risk levels in helping them make decisions about addressing their high LDLs? And to what extent are patients responding to their physician’s sense of urgency, versus to an understanding of their own health risks? The answers to these and many other questions could contribute substantially to future efforts to reduce population risks not only for LDL but for other conditions. Clalit and other health systems should promote evaluations of the pathways from provider to patient behavior in order achieve the high value, low waste care communities seek.

Despite this study’s demonstrated impacts on LDL levels, study results inevitably pose questions about how to enhance LDL impacts further, particularly among the highest risk group. For example, the reminders in this study targeted an LDL level of 100 for these high risk patients, while other research and health systems have targeted, for example, an LDL level of 70 for patients with prior coronary artery disease, as discussed in ATP III. Yet only 49 % of patients in the high risk group achieved the target goal, leaving the remaining half of the high risk population with a significant likelihood of potentially preventable arteriosclerosis-related deaths. 60 % of the medium and low risk groups achieved an identical target LDL of 100. The study thus raises questions about barriers to improvement in the high risk group, and about what interventions might best help this group. Further evaluation of the barriers to achieving LDL goals among the highest risk Clalit patients, and further testing of methods for overcoming them, is warranted.

Prior research on patients with chronic conditions suggests mental health concerns as a key driver of poor outcomes, especially among higher risk patients. A meta-analysis of interventions directed at improving hypertension care and outcomes pointed to stress, anxiety and depression as key barriers to adoption of a healthier lifestyle [6]. We know that patients at high risk of potentially preventable emergency department use or hospitalization disproportionately have mental health conditions [7]. A substantial proportion of the high risk high LDL patients who did not improve in relationship to the Clalit intervention are likely to belong to the overall high risk group of patients with multiple chronic diseases and mental health impediments. Such individuals are likely to be responsible for a disproportionate share of overall healthcare costs, making them especially critical for efforts to create higher value healthcare. Are different approaches needed to help these individuals achieve their best health outcomes? Is the availability in primary care of integrated mental health support or of additional support for lifestyle change needed for these individuals? Current information strongly suggests that more intensive intervention is needed for a substantial proportion of high risk patients.

As a related point, this study focused on physicians. Currently, in many systems, physicians are solely responsible for computer reminders. Yet primary care is moving ever more toward a more inclusive, patient-centered view that uses individual team members, including nurses and clerks at the core, often in combination with linked social workers, dietitians, mental health specialists and others [8]. Nurse practitioners may be particularly effective [9]. It would be helpful to know the extent to which additional team members were important in producing the observed improvements in this study. Furthermore, systematic approaches to involving non-physician healthcare professionals should be considered as a means for enhancing lipid management.

Finally, this study points to the large effects computer data views of the primary care population will have on how we practice medicine. In previous eras, medical personnel dealt with the patients in front of them. The computer data view asks us to consider the whole population, including those not seeking our care, or not willing or able to follow our guidelines. Medicine has always struggled with the tensions between the individual practitioner-to-patient interaction and the view of medicine as a major mediator of public health [10]. Healthcare professionals must discover how to resolve these tensions as the ability to view the full population and its outcomes becomes an increasing reality.

## Conclusions

In summary, the Clalit study is an exemplar for systematic assessment of a quality improvement by a healthcare delivery system. As such, it is part of the critical bridge between rigorous scientific studies, guidelines that synthesize them, and the implementation of the guidelines in clinical practice across a population. While each individual real world study like this provides a window into care, rather than a yes/no answer about intervention efficacy, this and other similar studies of science as applied to routine care are critical to the ongoing care improvement the public expects from its investments in scientific discovery.
